# Androgens regulate ovarian gene expression by balancing Ezh2-Jmjd3 mediated H3K27me3 dynamics

**DOI:** 10.1371/journal.pgen.1009483

**Published:** 2021-03-30

**Authors:** Sambit Roy, Binbin Huang, Niharika Sinha, Jianrong Wang, Aritro Sen

**Affiliations:** 1 Reproductive and Developmental Sciences Program, Department of Animal Sciences, Michigan State University, East Lansing, MI, United States of America; 2 Department of Computational Mathematics, Science and Engineering, Michigan State University, East Lansing, MI, United States of America; University of Pennsylvania, UNITED STATES

## Abstract

Conventionally viewed as male hormone, androgens play a critical role in female fertility. Although androgen receptors (AR) are transcription factors, to date very few direct transcriptional targets of ARs have been identified in the ovary. Using mouse models, this study provides three critical insights about androgen-induced gene regulation in the ovary and its impact on female fertility. First, RNA-sequencing reveals a number of genes and biological processes that were previously not known to be directly regulated by androgens in the ovary. Second, androgens can also influence gene expression by decreasing the tri-methyl mark on lysine 27 of histone3 (H3K27me3), a gene silencing epigenetic mark. ChIP-seq analyses highlight that androgen-induced modulation of H3K27me3 mark within gene bodies, promoters or distal enhancers have a much broader impact on ovarian function than the direct genomic effects of androgens. Third, androgen-induced decrease of H3K27me3 is mediated through (a) inhibiting the expression and activity of Enhancer of Zeste Homologue 2 (EZH2), a histone methyltransferase that promotes tri-methylation of K27 and (b) by inducing the expression of a histone demethylase called Jumonji domain containing protein-3 (JMJD3/KDM6B), responsible for removing the H3K27me3 mark. Androgens through the PI3K/Akt pathway, in a transcription-independent fashion, increase hypoxia-inducible factor 1 alpha (HIF1α) protein levels, which in turn induce JMJD3 expression. Furthermore, proof of concept studies involving *in vivo* knockdown of *Ar* in the ovary and ovarian (granulosa) cell-specific *Ar* knockout mouse model show that ARs regulate the expression of key ovarian genes through modulation of H3K27me3.

## Author summary

Androgens are traditionally viewed as the male hormone. Interestingly, in the last couple of years, androgens have emerged as a key regulator of fertility and health in women. High androgens are associated with polycystic ovary syndrome (PCOS) and is detrimental to women’s health. Conversely, androgen supplementation is now being used in clinical practice for a subgroup of women suffering from infertility as direct androgen actions are deemed essential for female fertility. However, in the ovary, which genes are regulated by androgens and how are poorly understood which is a limitation towards developing therapeutic or diagnostic strategies. We have discovered many important ovarian genes that were previously not known to be directly regulated by androgens. Additionally, we find that androgens influence the expression of a large number of ovarian genes critical for female fertility through epigenetic changes. Epigenetics govern whether a gene is “on” or “off” thereby affecting gene activity and expression. Here, we provide a mechanistic insight into how androgens decrease an epigenetic mark associated with gene repression. Our findings provide a comprehensive understanding of the overall impact of androgens on ovarian gene expression and physiology that can potentially improve female fertility and pathophysiological conditions like PCOS.

## Introduction

Androgens are traditionally considered as male hormones with well-established roles in male physiology and prostate cancer. However, in the last decade, several genetic models and *in vitro* studies have proven that androgens acting through androgen receptors (AR) are critical for ovarian function and female fertility [1–6]. While excess androgen level leads to polycystic ovary syndrome (PCOS) [7–9], a certain amount of direct androgen actions through the androgen receptor (AR) are essential for normal ovarian function [10]. Thus, it is now believed that with respect to androgen actions in the ovary, balance is key [4]. To date, in addition to the global androgen receptor knockout (ARKO) mouse models [11–13], AR has been knocked out specifically in different cell types along the hypothalamus-pituitary-gonadal (HPG) axis, namely granulosa cells (GCARKO) [14,15], theca cells (TCARKO) [16], oocyte (OoARKO) [15], pituitary (PitARKO) [17] and neurons (NeuroARKO) regulating the HPG axis [18]. All of these ARKO mouse models establish that the granulosa cells (GCs) of the ovary are the primary site of androgen actions in regulating normal follicular development and female fertility; while in hyperandrogenic conditions, neuroendocrine ARs play a major role in the development of PCOS [18]. Moreover, *ex vivo* [5,19], *in vitro* [20–23] and clinical studies [10,24–31] show that androgens are essential for follicle growth while simultaneously preventing follicular atresia. Despite these studies, how androgens regulate these follicular endpoints is poorly understood.

The major androgens in women, in descending order of serum concentration, are dehydroepiandrosterone sulphate (DHEAS), dehydroepiandrosterone (DHEA), androstenedione (A4), testosterone (T), and dihydrotestosterone (DHT) [32]. However, direct androgenic actions through the AR can only be mediated by T and DHT, as these are the only two androgens that bind to AR, the latter being more potent [33]. The rest of the androgens are more like androgen precursors that require conversion to T and/or DHT to exert androgenic effects. In the ovarian follicle and stroma, androgen synthesis primarily involves the conversion of cholesterol into pregnenolone (by CYP11A1) that is metabolized to DHEA (by CYP17A1) and then A4 (by 3β-HSD), the immediate precursor of testosterone [34]. A4 is converted into testosterone (17β-HSD), that can then be aromatized into estradiol (E_2_) by aromatase (CYP19a1) or reduced to DHT by 5α-reductase [34,35]. In women, 5α-reductase (*SRD5A1* and *A2*) mRNA expression has been found in both PCOS and non-PCOS women and interestingly, this expression is higher in granulosa cells than in theca cells [36,37]. In studies determining androgen actions in the ovary, DHT, which is non-aromatizable, is primarily used [5,19,38,39]. This is because T can be aromatized to estradiol, making it difficult to differentiate whether the downstream effects are due to estrogenic or androgenic actions, thereby confounding the results.

Androgen actions are mediated by *“nuclear”* transcriptional signals or *“extra-nuclear”* kinase actions [40–42]. Primary AR target genes are those at which AR occupies an androgen response element (ARE) on the promoter of a gene and regulates gene transcription. However, to date, very few ovarian genes have been identified as AR-ARE target genes and, intriguingly, there are no studies on the global impact of androgens on GC gene expression under normal conditions. Here we describe androgen-induced gene expression profiles in mouse GCs and provide molecular insight into the underlying mechanism of how androgens regulate the expression of these genes.

Importantly, this study also shows that androgens can regulate gene expression in an AR-ARE independent fashion, involving membrane-initiated androgen signaling [5,43–45]. Previously [38], we have reported that androgens influence gene expression through post-translational histone modifications. We have shown [38] that H3K27me3 (tri-methyl lysine 27 histone3) which is a gene silencing mark [46] is a downstream target of androgen actions. Here using ChIP-seq studies with H3K27me3 antibody we identify the ovarian (GC-specific) genes and their enhancer regions that are regulated by androgen-induced modulation of H3K27me3 mark. H3K27me3 is regulated by Enhancer of Zeste Homologue 2 (EZH2), a histone methyltransferase that promotes tri-methylation of lysine 27. Androgens, through both the extra-nuclear and nuclear pathways, inhibit the activity of EZH2 as well as *Ezh2* expression, respectively [38]. Epigenetic modulation of gene expression is dependent partly on the dynamic balance of histone methylation/demethylation on the enhancers and promoters. This is mediated by regulating the expression and activity of methylating and/or demethylating enzymes [47,48]. We demonstrate that in addition to inhibiting *Ezh2* expression and EZH2 activity, androgens also induce the expression of a histone demethylase called Jumonji domain containing protein 3 (*Jmjd3/Kdm6b*), that is responsible for removing the H3K27me3 mark. We find that in GCs, androgen in a transcription-independent fashion, increases hypoxia-inducible factor 1 alpha (HIF1α) protein levels, which in turn induce *Jmjd3* expression. This study not only provides a mechanistic understanding of the global impact of androgens in normal follicular development, but may also contribute to comprehending the effects of excess androgens as seen in disease conditions like PCOS.

## Results

### Effect of androgen on granulosa cell (GC) transcriptome

**Fig 1:**
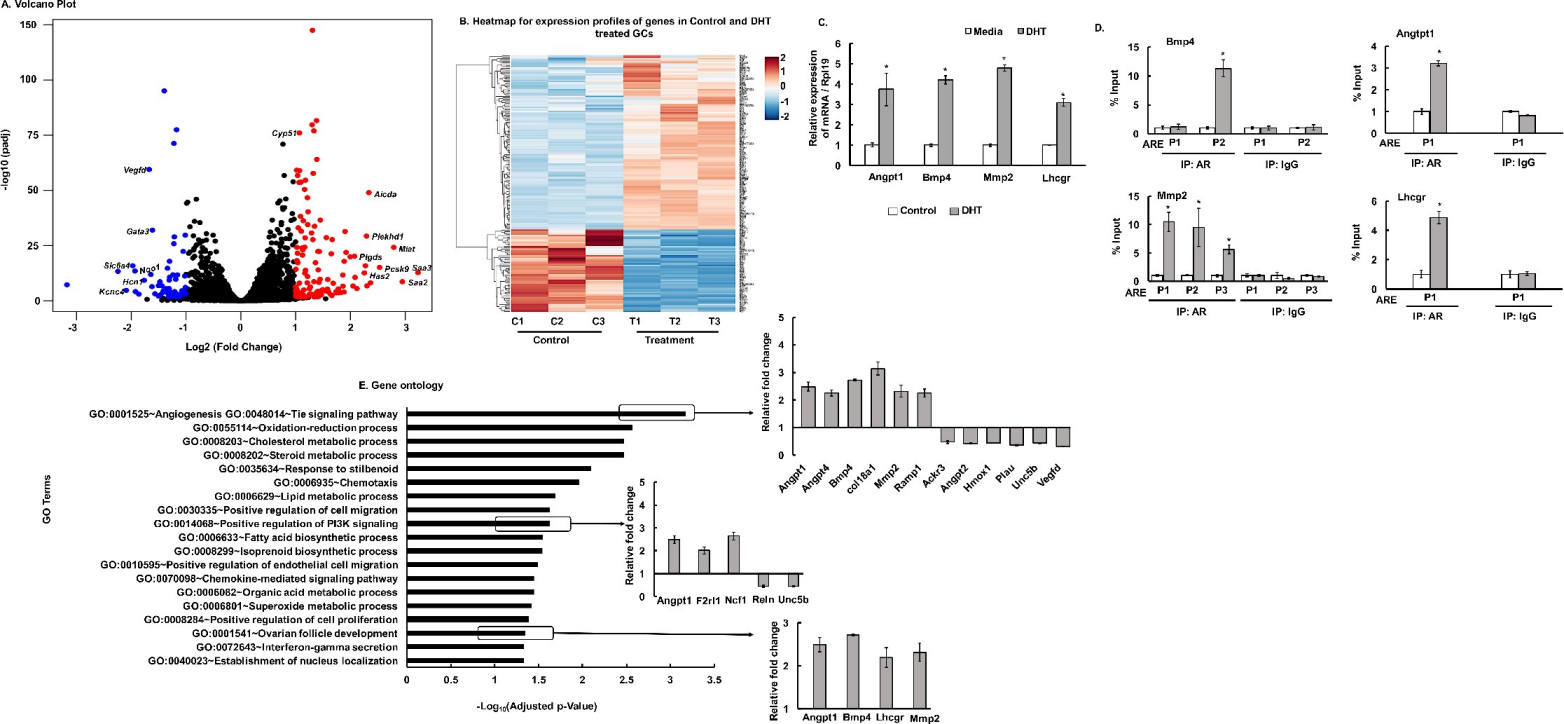
Androgen-induced transcriptome analysis in primary mouse granulosa cells (GC). A: Volcano plot: Representing the global transcriptional change across the groups compared. Each data point in the scatter plot represents a gene. Genes with an adjusted P ≤ 0.05 and a log2 fold change ≥ 1 are indicated by red dots and represent up-regulated genes. Genes with an adjusted P ≤ 0.05 and a log2 fold change ≤ -1 are indicated by blue dots and represent downregulated genes. B: Heatmap of differentially expressed genes sorted by adjusted p-value by plotting their log2 transformed expression values in samples. C: Relative expression of *Bmp4* (Bone morphogenetic factor 4), *Angpt1* (Angiopoietin 1), *Mmp2* (Matrix Metallopeptidase 2) and *Lhcgr* (Luteinizing hormone/Choriogonadotropin receptor) mRNA levels by quantitative PCR in primary mouse GCs treated with media or DHT (25nM for 24h). Data are displayed as means ± SEM (n = 3 experiments, for each experiment GCs isolated from 5 mice were pooled together) and normalized to *Rpl19* (* P ≤ 0.05, *vs* media). D: Anti-AR ChIP-assay in mouse primary GC cultures treated with media (control) or DHT (25nM for 24h) showing AR binding to different ARE sequences (P1/P2/P3) on *Bmp4* (-693/-383Kb from TSS), *Angpt1* (-1074Kb from TSS), *Mmp2* (-937/-753/-332Kb from TSS) and *Lhcgr* (-47Kb from TSS) promoter region. IgG represents non-specific antibody. Values represent percentage input. Data are displayed as mean ± SEM (n = 3 experiments, for each experiment GCs isolated from 10 mice were pooled together) and * P ≤ 0.05, *vs* media. E: Gene ontology analysis: Gene ontology terms of significantly enriched pathways with an adjusted P-value ≤ 0.05 in the differentially expressed gene sets.

Global effects of androgens in GCs were elucidated by RNA-seq analysis in primary mouse GC cultures treated with media (control) or DHT. The overall similarity among samples was assessed by the Euclidean distance between samples (S1 Fig). DESeq2 analysis identified a total of 190 annotated significant differentially expressed ENSEMBL genes (DEGs). Out of these genes, 129 were upregulated and 61 were downregulated genes (FPKM>1). The global transcriptional change across the two groups compared (control vs DHT) is represented by a volcano plot in Fig 1A and hierarchical clustering of all the significant DEGs in control vs DHT treated GCs are shown in Fig 1B. The complete list of significant DEGs is presented in S1 Dataset. *In silico* analysis revealed that all of the DEGs have at least one or more ARE sequences in the promoter and/or distal (within 5Kb) region thereby suggesting that most of these genes are regulated directly by AR, rather than secondary effects of hormone exposure. Based on established ovarian functions, 4 DEGs- *Bmp4*, *Angpt1*, *Mmp2* and *Lhcgr* were selected to further verify that AR, through direct ARE binding, regulates the expression of these genes. Fig 1C shows DHT treatment significantly increases mRNA abundance of *Bmp4*, *Angpt1*, *Mmp2* and *Lhcgr*. Moreover, ChIP-qPCR studies with AR antibody show that DHT treatment increases AR binding to AREs located on the promoter region of the above-mentioned genes (Fig 1D).

Gene ontology (GO) enrichment analysis (Fig 1E) of the DEGs revealed angiogenesis/tie pathway, cholesterol and lipid metabolism, steroid biosynthesis, oxidation-reduction process, ovarian follicle development and positive regulation of cell proliferation as some of the primary biological processes to be significantly affected by DHT treatment. Specific genes that are upregulated or downregulated in these pathways following androgen treatment are further shown in Fig 1E. These pathways/genes were previously not known to be regulated by androgens and, thereby, this study reveals that androgens affect a wide range of biological processes critical for normal follicular development and thus, ovarian function.

### Androgens significantly modulate H3K27me3 mark on gene promoters and enhancers

Previously [38] we have shown that androgens decrease the H3K27me3 mark in GCs. To evaluate the impact of androgens on genome-wide distribution of H3K27me3 landscape, we performed ChIP with the H3K27me3 antibody followed by high-throughput sequencing in control vs. DHT treated GCs.

#### Total H3K27me3 peaks modulated by DHT

**Fig 2:**
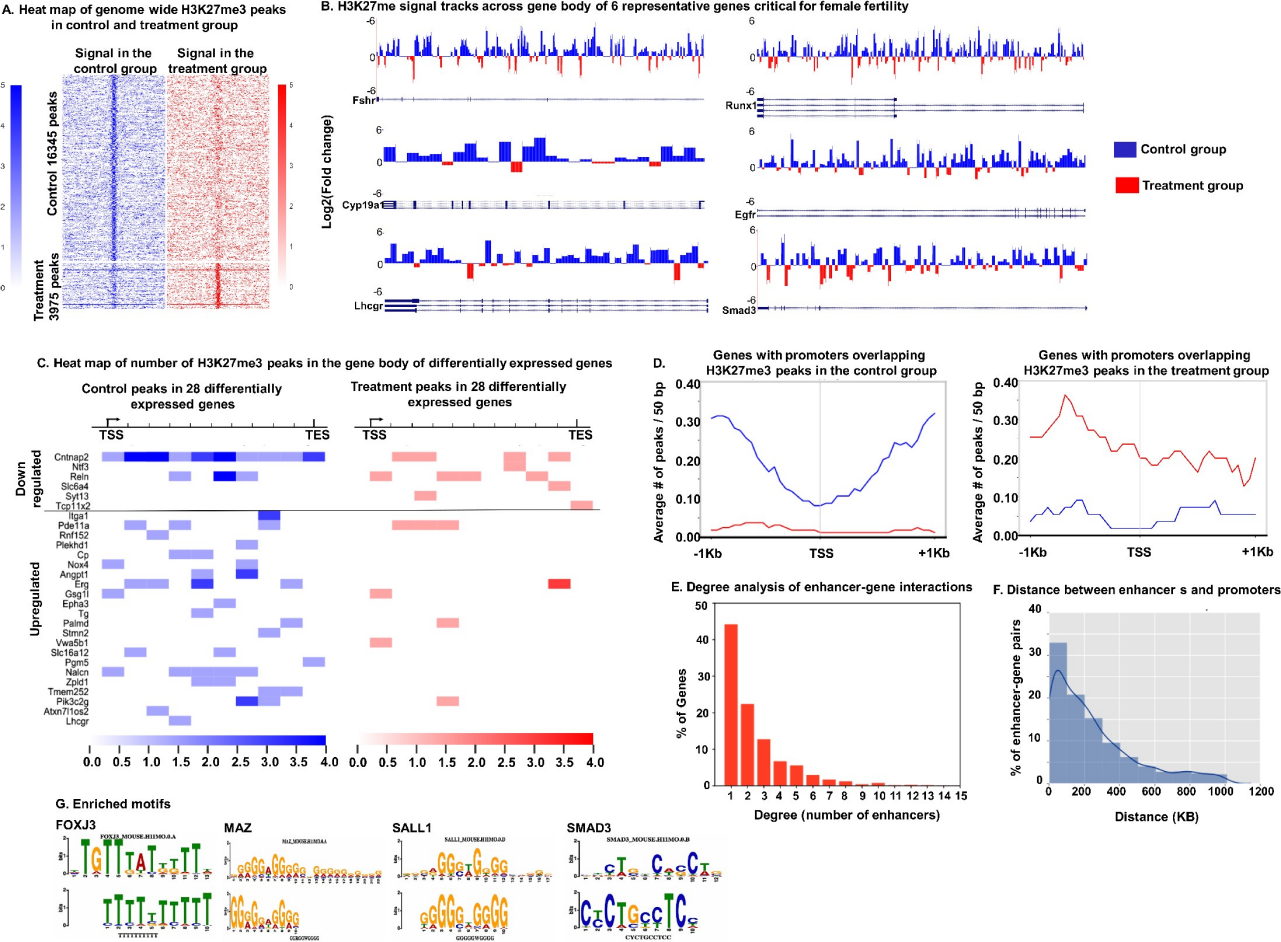
Genes associated with androgen-induced decrease in H3K27me3 mark in the gene body and overlapping with promoters and distal enhancers involving long-range regulation of gene expression. A: Heat maps showing the read density change along the peak regions for the 16345 control peaks and the 3975 treatment peaks. B: H3K27me3 signals in control and DHT treatment groups along the gene body of *Fshr-* follicle stimulating hormone receptor, *Cyp19a1-* aromatase, *Lhcgr-* luteinizing hormone/Choriogonadotropin receptor, *Runx1*- runt-related transcription factor 1, *Egfr*–epidermal growth factor receptor and *Smad3*- Mothers against decapentaplegic homolog 3. Log2 fold change was calculated as log2{(H3K27me3 control signal)/ (H3K27me3 treatment signal)}. Regions along the gene body with higher signals in the control group are represented as positive value- blue peaks and regions where signal was higher in the treatment group are shown as negative value-red peaks. C: Heatmap showing H3K27me3 peak counts in control and DHT-treated samples for 28 differentially expressed genes identified by comparing genes containing H3K27me3 peaks in the gene body with DEGs from the RNA-seq data. D: Average number of H3K27me3 peaks per 50bp overlapping with the promoter regions of 160 genes in the control samples and 55 genes in the DHT-treated samples. E: Degree analysis of enhancer-gene interactions for genes associated with significant decrease in H3K27me3 peaks in their enhancer regions with respect to DHT treatment. F: Distance between the promoters of genes and their corresponding enhancers that have decreased H3K27me3 signal by DHT treatment. G: Enriched transcription factor binding motifs in distal enhancers of genes associated with decrease in H3K27me3 signal with DHT treatment. E-values were < 0.1 and indicate the probabilities of observing the enrichment from random control DNA sequences. For each transcription factor, the upper motif logo corresponds to the consensus motif based on HOCOMOCO database and the lower motif logo corresponds to the observed sequence motifs that are enriched in linked distal enhancers.

The analysis of sequencing reads revealed 16,345 H3K27me3 peaks in control and 3975 H3K27me3 peaks in DHT treated samples: a 75% reduction in peaks in the DHT treated samples. Fig 2A shows a heat map of genome wide H3K27me3 peaks in GCs from control and treatment groups. Each row in the heatmap corresponds to a H3K27me3 signal peak identified from either controls or DHT treated samples. The normalized ChIP-seq read densities around the peaks are shown with the summits of the peaks in the middle, along with +/-10kb flanking regions. Of 16,345 H3K27me3 peaks in control samples, 5513 peaks were within gene bodies while 153 peaks were in promoter regions. In contrast, there were only 1389 H3K37me3 peaks in the gene body and 54 peaks in the promoter region in the DHT treated samples. S2 Fig represents the number of H3K27me3 peaks overlapping different genomic annotations in the control and treatment (DHT) group.

#### DHT-induced modulation of H3K27me3 peaks in gene bodies

To further analyze the influence of androgen-modulated epigenetic dynamics on genes, we examined genes with gene bodies overlapping with H3K27me3 peaks. We identified 3144 genes in control and 1146 genes in DHT treated GCs with H3K27me3 peak signal across the gene body. Comparison of these two gene sets revealed that 2462 genes exclusively had H3K27me3 peak signal across the gene body in the control but not in the DHT-treated samples (S2 Dataset). Fig 2B demonstrates log2-fold change of H3K27me3 peaks overlapping with gene bodies of six representative genes (*Fshr*, *Cyp19a1*, *Lhcgr*, *Runx1*, *Egfr* and *Smad3*) that are known to play critical roles in ovarian function. For each gene, the H3K27me3 mark in control and treatment signals along the gene body was calculated by dividing each gene into 1000bp windows. The number of reads falling under each 1000bp window were considered the H3K27me3 signal in that window and log2 fold change of H3K27me3 signals along the gene body was calculated. Results show that DHT-treatment significantly lowers the H3K27me3 signal in all of the genes.

Subsequently, we compared the list of genes containing H3K27me3 peaks in their gene bodies with the list of DEGs from the RNA-seq data. We found 28 genes (22-upregulated and 6-downregulated genes) that were both differentially expressed and overlapped with H3K27me3 peaks, suggesting that the condition-specific epigenetic landscape of H3K27me3 may be related to the transcriptional variation of these genes. Fig 2C is a heat map representing the number of H3K27me3 peaks in the gene body (TSS to TES) for the 28 DEGs. Most of the upregulated genes had significantly lower levels of H3K27me3 marks while the downregulated genes had higher H3K27me3 marks in DHT-treated GCs than in controls. This shows that in addition to AR-ARE, the expression of these genes may also be regulated by androgen-induced H3K27me3 modulation.

#### DHT-induced modulation of H3K27me3 peaks in the promoter region

Further analysis revealed that there were 160 genes in control and only 55 genes in the DHT treated samples with H3K27me3 peaks located specifically in the gene promoter regions. Fig 2D (Left panel) represents the average number of H3K27me3 peaks per 50bp in the promoter region (TSS+/-1KB) for the 160 genes in the control group and corresponding average peaks for the same genes in the treatment group. Similarly, Fig 2D (right panel) represents the average number of H3K27me3 peaks per 50bp in the promoter region (TSS+/-1KB) for the 55 genes in the treatment group and corresponding average peaks for the same genes in the control group. The list of all the genes with promoters overlapping with H3K27me3 peaks in the control and treatment groups is provided in the supplemental data (S3 Dataset). Considering the complex regulatory activities in promoter regions, these androgen-induced differential H3K27me3 peaks located in the promoters may play pivotal roles in regulating the transcriptional levels of the corresponding genes.

#### DHT-induced modulation of H3K27me3 peaks in the enhancer region

Since large portions of the mouse genome are non-coding regions with numerous enhancers widely spread [49], we further extended our analysis to non-coding regions and focused on distal enhancers that have long-range chromatin interactions with promoters and may play a crucial role in controlling the expression of genes. While enhancers act through the binding of transcription factors just like promoters, their location greatly vary from the transcription start site (TSS) of the gene they regulate. Moreover, while a single enhancer can influence the expression of multiple genes, a single gene can be regulated by multiple enhancers. Thus, we determined enhancer-gene pairs for the 2462 genes in which the H3K27me3 signal peaks were significantly decreased by DHT treatment (genes with exclusive H3K27me3 peaks in the control group). For these genes, the chromatin interaction data including Hi-C and Capture-C were used to find potential enhancers (Bioinformatics analysis for ChIP-seq, S1 Text). For each gene, the H3K27me3 in the gene body and H3K27me3 level in each of its potential enhancers were calculated. We found 1380 genes where DHT treatment lowered the H3K27me3 signal (S5 Dataset). For enhancer–gene pair, the correlation between the gene body H3K27me3 level and enhancer H3K27me3 level was calculated and only positively correlated enhancer–gene pair (Pearson correlation > 0.4) were selected. Results show 3447 enhancer–gene pairs. Next, we determined the number of enhancers that regulate the same gene (Fig 2E). Results show that 45% of these genes are regulated by only 1 enhancer region while 21% of the genes are regulated by 2 enhancers. Furthermore, we calculated the distance between the promoters and their corresponding enhancers that have decreased H3K27me3 signal by DHT treatment. Fig 2F shows the distance analysis of the enhancer–gene interactions for the genes that show DHT-induced decrease in H3K27me3 levels. 33% of the enhancer–gene pairs that show decreased H3K27me3 with DHT treatment have 0 to 100KB distance between the gene and the enhancer. These analyses show that androgens can modulate gene expression not only by reducing the H3K27me3 mark in the promoter region of genes but also in distal enhancers.

Moreover, comparing the chromatin contact maps (ChIP-seq data) with the androgen-induced DEGS revealed 186 enhancers whose H3K27me3 levels were significantly negatively correlated (Pearson correlation < -0.4) with the expression of 99 DEGs, out of which 66 were upregulated and 33 were downregulated genes (S6 Dataset) across samples of controls and DHT treated GCs. This highlights the importance of complex long-range multi-enhancer regulation of AR regulated genes in the ovarian GCs.

#### Motif analysis of the enhancers

Given that H3K27me3 is a gene repressive mark, it is likely that the androgen-induced decrease of H3K27me3 allows specific transcription factors to bind to these enhancer regions. We analyzed the enhancer regions linked with H3K27me3 peaks for motif enrichment using MEME-ChIP as described in bioinformatics analysis for ChIP-seq, (S1 Text). Four transcription factors (TFs), FOXJ3 (forkhead box j3; *p*-value 1.44e-04 and *q*-value 1.49e-01), MAZ (MYC associated zinc finger protein; *p*-value 6.85e-08 and *q*-value 7.05e-05), SALL1 (spalt like transcription factor 1; *p*-value 2.20e-04 and *q*-value 9.90e-02) and SMAD3 (*p*-value 3.20e-05 and *q*-value 3.32e-02) with E-value < 0.1 were identified as candidate factors associated with epigenetic changes in distal enhancers (Fig 2G).

### Androgens through the PI3K/Akt pathway, in a transcription-independent fashion, increase HIF1α protein levels, which in turn induce the expression of JMJD3 / KDM6B in mouse granulosa cells

**Fig 3:**
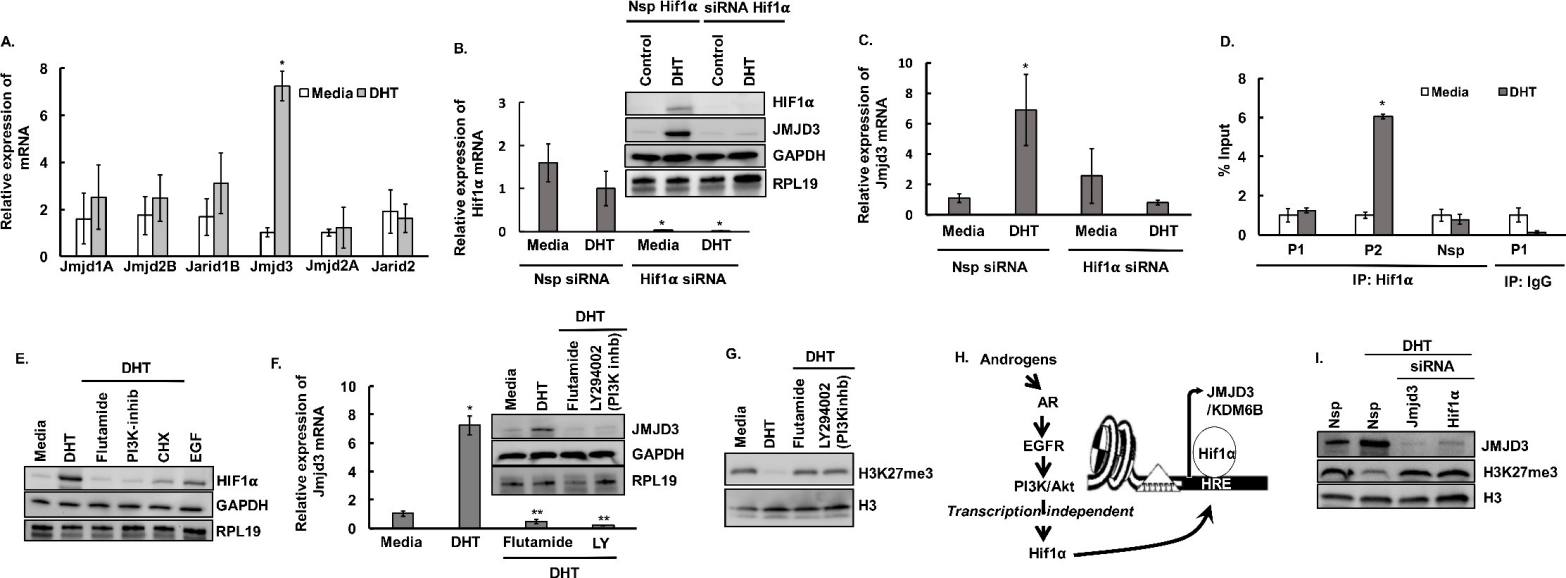
Androgens through HIF1α regulate JMJD3 expression. A: Relative expression of Jumonji histone demethylase mRNA levels by quantitative PCR in mouse primary GC cultures treated with media containing vehicle (control) or DHT (25nM for 24h). Data are displayed as mean ± SEM (n = 3 experiments, for each experiment GCs isolated from 5 mice were pooled together) and normalized to *Rpl19* (* P ≤ 0.05, *vs* media). B: siRNA-mediated knockdown of *Hif1α* in mouse primary GC cultures. C: Effect of siRNA-mediated knockdown of *Hif1α* on *Jmjd3* mRNA levels in mouse primary GC cultures treated with media containing vehicle (control) or DHT. Data are displayed as mean ± SEM (n = 3 experiments, for each experiment GCs isolated from 5 mice were pooled together) and normalized to *Rpl19* (* P ≤ 0.05, *vs* media). D: Anti-HIF1α ChIP-assay in mouse primary GC cultures treated with media containing vehicle (control) or DHT (25nM for 24h) showing HIF1α binding to two (P1/P2) HRE sequences (-0.2/+4.2Kb from TSS) on *Jmjd3* promoter region. IgG represents non-specific antibody and “Nsp” represents non-specific primers. Values represent percentage input. Data are displayed as mean ± SEM (n = 3 experiments, for each experiment GCs isolated from 10 mice were pooled together) and * P ≤ 0.05, *vs* media. E: HIF1α protein levels in mouse primary GC cultures treated with media containing vehicle (control) or DHT (25nM for 24h) in presence of flutamide (AR inhibitor, 100nM), LY294002 (PI3K inhibitor, 10μM) or cycloheximide (CHX, a protein translational inhibitor, 1μM). Epidermal Growth Factor, EGF (20ng/ml) was used as a positive control. F and G: Relative expression of *Jmjd3* mRNA and protein levels (F) and H3K27me3 levels (G) in mouse primary GC cultures treated with media containing vehicle (control) or DHT (25nM for 24h) in presence of flutamide (AR inhibitor, 100nM) and LY294002 (PI3K inhibitor, 10μM). Data are displayed as mean ± SEM (n = 3 experiments, for each experiment GCs isolated from 5 mice were pooled together) and normalized to *Rpl19* (P ≤ 0.05, * *vs* media and ** *vs* DHT). H: Proposed model of androgen-induced translation-dependent increase in HIF1α protein levels and its role in *Jmjd3* expression. I: Effect of siRNA-mediated knockdown of *Hif1α* and *Jmjd3* on H3K27me3 levels in mouse primary GC cultures treated with media containing vehicle (control) or DHT (25nM for 24h).

Next, we determined the underlying mechanism by which androgens modulate H3K27me3. We have reported [38] that androgens inhibit the expression and activity of the histone methyltransferase EZH2, that promotes the H3K27me3 epigenetic mark. Here we wanted to determine if androgens have any effect on the expression of histone demethylases. Results (Fig 3A) show that 24h of DHT (25nM) treatment specifically induces the expression of a Jumonji domain containing demethylase called *Jmjd*3 (*Kdm6b*). There was no effect of DHT on the expression of other Jumonji domain containing demethylases namely, *Jmjd1a (Kdm3a)*, *Jmjd2b (Kdm4b)*, *Jarid1b (Kdm5b)*, *Jmjd2a (Kdm4b)* and *Jarid2*. Intriguingly, *Jmjd*3 specifically demethylases H3K27me3 and therefore we further explored the mechanism by which androgens regulate the expression of *Jmjd*3.

*In silico* analysis of the *Jmjd*3 promoter revealed the presence of HRE (*Hif1α* response element) regions (S3A Fig). Interestingly, studies in mouse and humans show that HIF1α can induce *Jmjd*3 both under normal conditions and hypoxic stress [50]. Therefore, to determine if HIF1α plays any role in DHT-induced expression of *Jmjd*3 in mouse GCs, we knocked down the expression of *Hif1α* with *Hif1α* -specific siRNA (Fig 3B) in primary mouse GC culture. siRNA-mediated knockdown of *Hif1α* completely blocked the DHT-induced increase of *Jmjd*3 mRNA (Fig 3C) and JMJD3 protein levels (Fig 3B) in mouse GCs. Furthermore, ChIP studies with HIF1α antibodies (Fig 3D) show increased binding of HIF1α on one of the two HRE sequences on the *Jmjd*3 promoter region with DHT treatment. This establishes that androgens, through HIF1α, mediate the expression of *Jmjd3* in mouse GCs.

We further wanted to understand how androgens regulate HIF1α. In prostate cancer, it has been reported that androgens acting through the PI3K/Akt pathway, in a transcription-independent fashion, increase HIF1α protein levels [51]. Consequently, we and others have also reported that DHT treatment activates the PI3K/Akt pathway in GCs [6,19,38]. Results show that similar to prostate cancer, in mouse GCs, DHT stimulation does not affect *Hif1α* mRNA levels (Figs 3B and S3B) but increases HIF1α protein levels (Fig 3B and 3E). This increase in HIF1α protein by DHT stimulation is blocked by flutamide (AR inhibitor), LY294002 (PI3K inhibitor) and cycloheximide (CHX, a protein translational inhibitor) (Fig 3E). Moreover, CHX-chase studies with DHT revealed no difference in HIF1α protein degradation (S3C Fig), indicating that DHT increases HIF1α protein levels through a translation-dependent mechanism. Notably, inhibition of AR and the PI3K pathways attenuated DHT-induced increase in *Jmjd3* mRNA (Fig 3F), JMJD3 protein levels (Fig 3F) and JMJD3 enzymatic activity (S3D Fig) as well as the global decrease in the H3K27me3 mark (Fig 3G) in mouse GCs. Thus, these studies show that androgens, through PI3K/Akt signaling, in a translation-dependent pathway enhance HIF1α protein levels that in turn bind to HRE sequences on the *Jmjd3* promoter region and induce the expression of *Jmjd*3 in mouse GCs (Fig 3H).

Furthermore, to directly establish the AR-HIF1α-JMJD3 pathway to the downstream modulation of H3K27me3 mark, we knocked down *Jmjd3* and *Hif1α* by siRNAs and determined H3K27me3 levels (Fig 3I), as well as performed ChIP assays with H3K27me3 antibody on *Lhcgr*, *Cyp19a1*, *Fshr and Runx1* promoter regions (S4A Fig). Results show that ablation of *Jmjd3* and *Hif1α* expressions block the androgen-induced decrease of total H3K27me3 levels (Fig 3I) and the H3K27me3 mark in the promoter region of the above-mentioned genes (S4A Fig).

Previous studies have shown that hypoxia alone or Clioquinol, a *Hif1α* activator under normoxia, can induce *Jmjd3* expression [50]. Therefore, to determine whether exogenous activation of HIF1α in absence of AR can rescue *Jmjd3* expression and H3K27me3 levels, we knocked down *Ar* by LNA-siRNA in primary mouse GC cultures and treated the cells with/without Clioquinol (50μM) in presence or absence of DHT (S5 Fig). Similar to previous studies, Clioquinol induces JMJD3 protein and lowers H3K27me3 level in mouse primary GCs (S5A Fig). However, in absence of AR, while Clioquinol completely rescues JMJD3 level, the downstream effect on H3K27me3 level (decrease of H3K27me3) was only partial (S5B Fig). This suggests that androgen-induced modulation of the H3K27me3 mark is mediated through a synergistic regulation of the expression and activity of the two opposing enzymes, EZH2 and JMJD3 that specifically target tri-methylation of H3K27. In addition to H3K27me3 levels, we also determined H3K27ac levels, a well-recognized marker for active enhancers. H3K27ac levels were significantly elevated in GCs treated with DHT compared to controls (S4B Fig).

### Androgen-induced modulation of H3K27me3 regulates expression of genes critical for ovarian function

**Fig 4:**
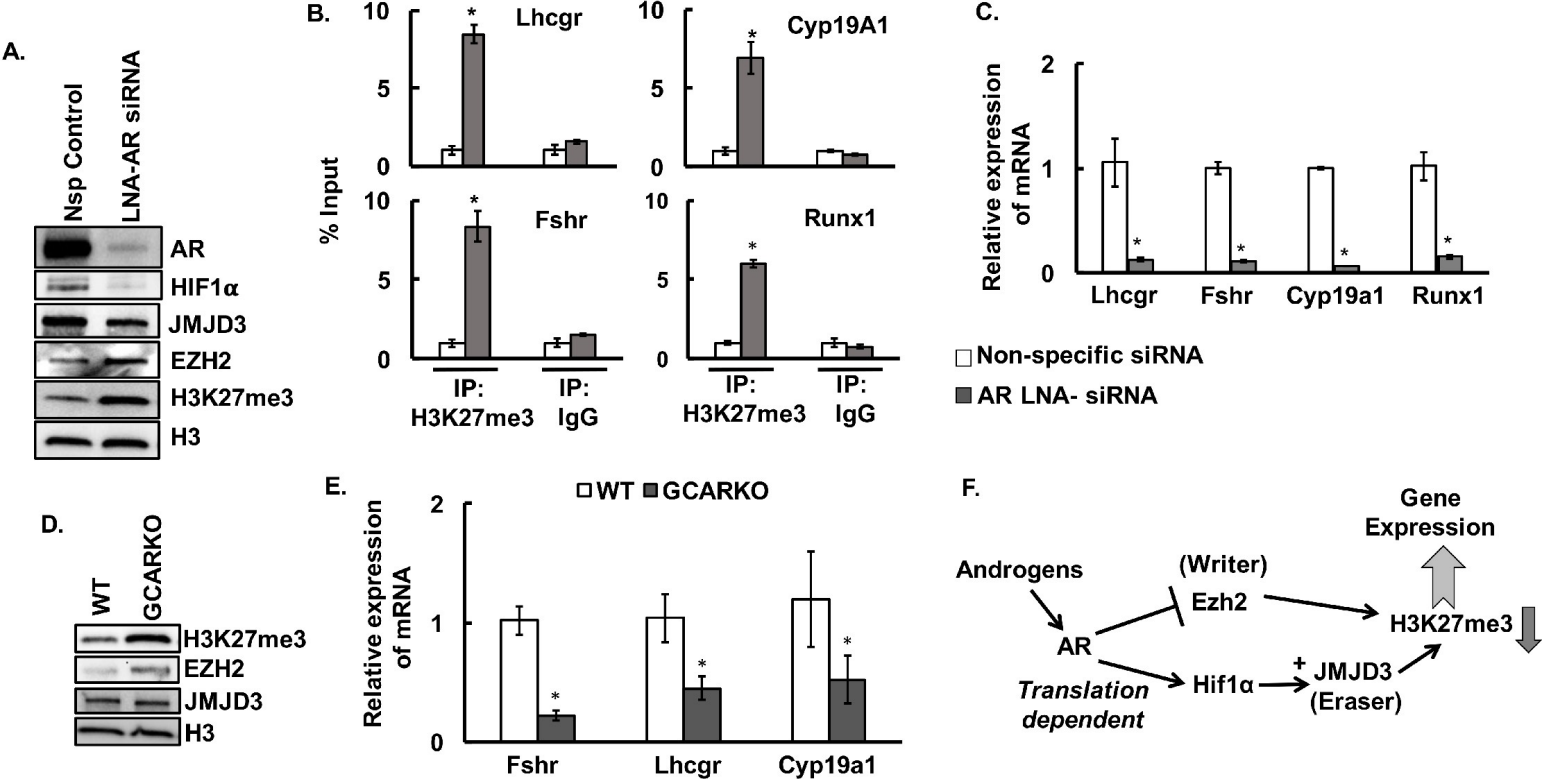
*In vivo* knockdown of androgen receptors increases H3K27me3 mark and inhibits the expression of key ovarian genes. A: Protein levels of androgen receptor (AR), HIF1α, JMJD3, EZH2, H3K27me3, and H3 in GCs isolated from ovaries of mice injected in the ovarian bursa with LNA-locked *Ar* siRNA and non-specific (Nsp) siRNA control. B and C: Anti-H3K27me3 ChIP-assay showing H3K37me3 levels within the gene body of *Lhcgr* (+641/+740 bp from TSS), *Fshr* (+379/+488 bp from TSS), *Cyp19a1* (+553/+653 bp from TSS) and *Runx1* (+579/+693 bp from TSS) (B) and relative expression of mRNA of these genes (C) in GCs isolated from ovaries of mice (n = 15 mice) injected in the ovarian bursa with LNA-locked *Ar* siRNA and non-specific (Nsp) siRNA. IgG represents non-specific antibody. ChIP assay values represent percentage input and data are displayed as mean ± SEM (n = 3 ChIP assays, for each experiment GCs isolated from 5 mice were pooled together) and *P ≤ 0.05, *vs* non-specific siRNA. Relative expression of mRNA is displayed as mean ± SEM (n = 3 experiments, for each experiment GCs isolated from 5 mice were pooled together) and normalized to *Rpl19* and *P ≤ 0.05, *vs* non-specific siRNA. D and E: H3K27me3, EZH2, JMJD3 and H3 protein levels (D) and relative expression of *Fshr*, *Lhcgr* and *Cyp19a1* mRNA levels (E) in GCs isolated from ovaries of 24-25-week-old GC- specific ARKO (GCARKO) and wild type (WT) mice. Data are displayed as mean ± SEM (n = 4 mice) and normalized to *Gapdh* (*P ≤ 0.05 *vs* WT). F: Proposed model of androgen-induced modulation of H3K27me3 through regulation of *Ezh2* and *Jmjd3* expression and activity.

Finally, as a proof of concept, we knocked down AR expression *in vivo* by injecting LNA-containing oligonucleotides targeting AR or non-specific control into the ovarian bursa of mice (Fig 4A). Loss of AR expression *in vivo* significantly increases the H3K27me3 mark in the GCs that corresponds to increased expression of EZH2 and decreased levels of HIF1α and JMJD3 than in non-specific controls (Fig 4A). ChIP-PCR studies (Fig 4B) with H3K27me3 antibody also reveal higher level of H3K27me3 within the gene body of key ovarian genes, *Lhcgr*, *Fshr*, *Cyp19a1* and *Runx1*. The downstream effect of increased H3K27me3 levels was further reflected by decrease in the mRNA levels of *Lhcgr*, *Fshr*, *Cyp19a1* and *Runx1* in the GCs treated with LNA-AR siRNA compared to non-specific control (Fig 4C).

To further prove that the androgen/AR-induced modification of H3K27me3 is physiologically important, we determined H3K27me3 levels and expression of *Lhcgr*, *Fshr* and *Cyp19a1* in GCs isolated from ovaries of 24-25-week-old GC-ARKO mice. Notably, we have shown previously [15,38] that GC-specific knockout of AR causes premature ovarian failure characterized by higher rate of atresia and fewer ovulated oocytes. Based on our present data it can be speculated that one of the reasons for the premature ovarian failure in the GC-ARKO mice is due to the loss of androgen-induced decrease in H3K27me3 mark that in turn regulates the expression of many key ovarian genes. Supporting this hypothesis, results show that knockout of ARs, specifically in the GCs of the ovary, indeed increases H3K27me3 and EZH2 levels (Fig 4D) with decrease in JMJD3 protein levels. Moreover, expression of *Lhcgr*, *Fshr and Cyp19a1* are significantly lower (Fig 4E) in the GCs isolated from the ovaries of GC-ARKO animals compared to wild type.

## Discussion

In the ovary, androgens are not merely a substrate for estrogen synthesis, but direct androgen actions through the ARs are critical for normal follicular development and female fertility [2,4]. However, there is a dearth of knowledge about the genes and biological pathways regulated by androgens, which is a significant limitation towards understanding how androgens regulate follicular growth and function. This study for the first time provides three critical insights about androgen actions in the ovary.

First, we have identified a large number of genes and biological processes that were not formerly known to be regulated directly by androgens in GCs. Results show that genes like *Bmp4* [52], *Lhcgr* [53], *Adamts4* [54], *Ptgds4* and *Mmp2* [55–57], that are critical for follicular function are AR-induced genes. Previously, we [5] and others [6,19] have reported that androgens primarily maintain normal follicular development by regulating pre-antral to antral follicle transition by increasing FSH receptor levels and prevent follicular atresia. However, our gene expression data now clearly show that androgens have a much far-reaching impact on follicular function. For example, the angiopoietin-tie pathway, many of the genes of which we find to be directly induced by androgens, are not only vital for the remodeling and maturation of the developing vasculature that helps in the formation of dominant follicle/s but also play an essential role in non-angiogenic functions like follicular survival and steroidogenesis [58,59]. Inhibition of ANGPT1 increases follicular atresia [60] and the follicles which undergo early atresia show a higher ANGPT2:ANGPT1 ratio [61–63]. This is consistent with our results—DHT treatment increases *Angpt1* expression and lowers *Angpt2* transcript levels. ANGPT2 is considered a natural antagonist of ANGPT1 [64,65]. The latter induces the phosphorylation of Tie2, which subsequently transduces a biological effect while ANGPT2 binds to Tie2 with the same affinity as ANGPT1 but does not phosphorylate the receptor [64,65]. Therefore, it is likely that the androgen-induced regulation of the angiopoietin–tie pathway is another mechanism by which androgens prevent follicular atresia. Notably, the fact that androgen treatment directly induces genes in the cholesterol-lipid metabolism, steroid biosynthesis and oxidation-reduction pathways, which are some of the basic biological processes involved in maintaining normal follicular development [66–68], further highlights the importance of androgens in follicular development. Given that preovulatory androgens are essential for normal ovarian function and female fertility, while excess androgen is one of the well-established underlying causes for PCOS, we propose that there exists a critical balance between the essentiality of androgens for normal follicular function and the detrimental effects seen in hyperandrogenic conditions. For example, a number of genes like *Lhcgr* [69], *Angpt1* [70] and *Mmp2* [71], that were upregulated following androgen treatment (RNA-seq data), not only play critical role in follicular development but have also been shown to be elevated in PCOS patients.

Moreover, androgen treatment also resulted in downregulation of 61 genes. Interestingly, comparison of the RNA-seq and ChIP-seq dataset revealed that out of all the downregulated genes, only 6 genes had higher H3K27me3 mark (Fig 2C). This suggests that androgen-induced downregulation of genes may be a secondary effect of androgen treatment. For example, we have reported previously [38] that androgens induce the expression of *miR-101* that in turn downregulates the expression of *Ezh2*. Another example is androgen-induced expression of *miR-125b* that decreases the expression of pro-apoptotic proteins [5].

Second, we have performed ChIP-seq analysis in GCs to determine the DHT-induced changes in H3K27me3, which is a gene silencing mark. Control of gene expression is exerted at a number of levels, one of which is the accessibility of genes and their controlling elements to the transcription machinery. Accessibility is dictated broadly by the degree of chromatin compaction, which is influenced in part by post-translational histone modifications. Our results highlight an important concept: in GCs, in addition to the genomic actions of androgens through the “classical” AR-ARE binding, androgen-induced decrease in H3K27me3 mark is another avenue through which androgens can regulate gene expression. The fact that we identified only 190 DEGs (from RNA-seq study) that are directly regulated by androgens in contrast to 2462 genes (from ChIP-seq) that specifically had lower H3K27me3 mark in DHT-treated GCs, clearly shows that androgen-induced modulation of H3K27me3 mark has a much broader impact than the direct effects of androgens on GC function. On the basis of our present study, we propose that in GCs, androgens prime the promoter and/or enhancer regions of genes by lowering the H3K27me3 mark, that enables other transcription factors to induce the expression of these genes. In fact, we have reported previously [38] that *Runx1*, a gene critical for ovulation, is one such downstream target and androgens remove the H3K27me3-repressive mark from the *Runx*1 promoter. This enables the hCG-induced transcription machinery to access the *Runx1* promoter region leading to increased expression of *Runx1*. Intriguingly, previous studies have reported that androgen treatment increases the expression of genes like *Fshr* [6,72–75] and *Cyp19a1* [1,76,77] that are critical for follicular development. However, there was no evidence that these genes are direct targets of AR-ARE mediated actions. Prior to this study, it was not known how androgens regulated the expression of these critical ovarian genes to promote ovarian function and female fertility, in general. We now show that androgen treatment significantly lowers the H3K27me3 mark in the gene body of *Fshr* and *Cyp19a1* which provides a mechanistic explanation of how androgens, independent of AR-ARE interaction, through H3K27me3 modulation may influence the expression of these genes. Moreover, some of the genes with lower H3K27me3 mark on the gene body/enhancer regions following DHT treatment, like *Cyp19a1* [78], *Adamts15* [79] *Casp7* [80], *Erbb4* [81] and *Lepr* [82] have been reported to be elevated and/or associated with PCOS.

Third, our studies highlight that in GCs, androgens modulate the H3K27me3 mark by balancing the expression and activity of two opposing epigenetic enzymes, EZH2 and JMJD3. While on one hand androgens inhibit EZH2 [38], which causes the tri-methylation of H3K27, androgens also induce the expression of *Jmjd3*, which is a histone demethylase that specifically removes the tri-methylation mark from H3K27 (Fig 4F). Moreover, in addition to the ‘*genomic*’ effects of AR, it is well-established that androgens can also induce transcription-independent ‘*non-genomic*’ effects [40–42,45]. Our studies not only establish HIF1α as one of the downstream targets of non-genomic androgen actions, but also demonstrate that androgens indirectly induce the expression of *Jmjd3* in GCs. Studies [83] in rat models show that *Hif1α* is expressed in the GCs throughout follicular development (from primary to pre-ovulatory follicles). Moreover, in pre-ovulatory follicles, *Hif1α* gene expression has been reported to be regulated by the actions of progesterone–progesterone receptor [84]. It is possible that dependent on the stage of follicular development, HIF1α may be regulated by different steroid hormones. For example, based on AR expression level at different follicular stages [1,85] as well as studies on androgenic effects during follicular development [4], it is believed that AR-mediated actions are predominantly in the pre-antral and small antral follicles and these effects diminish as the follicle develops to large antral/pre-ovulatory stage. Therefore, it can be speculated that in the pre-antral and small antral stages, androgens regulate HIF1α protein levels and following the LH surge, progesterone becomes the primary regulator of *Hif1α*. Further studies are needed to establish this hypothesis. Given that androgens increase HIF1α protein levels, it is likely that in addition to *Jmjd3* expression, other HIF1α-target genes also get induced following androgen treatment. Intriguingly, a large number of upregulated genes in our RNA-seq data are HIF1α target genes. For example, *Bmp4* [86,87], *Angpt1* [88], *Mmp2* [89,90], *Has2* [91], *Pcsk9* [92], *Erg* (ETS transcription factor) [93,94] and *Ifitm1* [95] are some of the genes that have previously been reported to be HIF1α target genes. Studies [96] in cancer cells have reported significant crosstalk between AR and HIF1α. Co-immunoprecipitation assays have confirmed a direct interaction between AR and HIF1α, and ChIP analysis showed HIF1α interacts with the AR in genes involved in prostate cancer [96]. Similar interactions may also occur in the GCs.

Interestingly our studies also show that in addition to lowering the gene repressive H3K27me3 mark, androgens increase the gene activating H3K27ac mark. This is in accordance with previous studies that show reciprocal changes of H3K27ac and H3K27me3 modifications at the promoter regions of genes [97,98]. Moreover, EZH2 (inhibited by androgens) may function as an “on-off” switch, that acts not only as a critical epigenetic repressor, but also as an activator for specific downstream targets [99]. The EZH2 molecular switch functions mainly between H3K27me3 repressive mark and H3K27ac positive mark. EZH2 antagonizes CBP/p300 actions (primary enzyme for acetylation of lys-27 on H3) and inactivation or decrease of EZH2 increases CBP/p300-mediate acetylation of H3K27 [98,100]. Therefore, it is possible that in GCs, androgens by inhibiting *Ezh2* and inducing *Jmjd3* expression lower H3K27me3 mark and also simultaneously increase the H3K27ac mark. Further studies are needed not only to establish this hypothesis but also to determine the H3K27ac pattern with respect to gene expression in GCs following androgen treatment.

In summary, given the role of androgens in female fertility and women’s health in general, results of this study provide a global perception of androgen effects in follicular function and insights into the androgen-induced molecular mechanisms responsible for normal ovarian physiology as well as for disease conditions like PCOS.

## Materials and methods

Details of all experimental methods are provided in the supplemental information (S1 Text).

### Ethics statement

Mouse studies were performed in accordance with the guidelines for the care and use of laboratory animals and were approved by the Institutional Animal Care and Use Committee (IACUC) at MSU under the approval number PROTO202000156.

### Animals and cell culture

Unless otherwise mentioned, mouse experiments were performed in 8–9 week old C57BL/6J mice (The Jackson Laboratories). Collection and culture of mouse GCs were performed as described previously [5,38,44,98,101–103]. Different concentrations of DHT (5nM, 10nM and 25nM) were used to determine the effect of androgen on HIF1α, JMJD3 and H3K27me3 levels (S6A Fig) as well as gene expression of *Bmp4*, *Angpt1*, *Lhcgr and Mmp2* (S6B Fig). All DHT concentrations showed similar results and based on our previous studies [38,45] 25nM DHT concentration was selected. DHT instead of testosterone was used in order to avoid misinterpretation of results due to aromatization of testosterone to estradiol.

### siRNA knockdown experiments

siRNA-mediated knockdown experiments in primary mouse GCs [5,98,103] and ovarian bursal injections [5,38] were performed as previously described.

### Chromatin immunoprecipitation (ChIP) assay

ChIP was performed as previously described [5,38,98,103,104] and List of ChIP primers (S4 Dataset) and antibodies (S7A Fig) are provided in the supplemental data.

### RNA isolation, RNA-seq and Bioinformatics analysis

Total RNA isolation, library construction and RNA-sequencing services were carried out by Genewiz, Inc. Details of the RNA isolation, RNA-seq and bioinformatics analysis of RNA-seq data are provided in the supplemental material.

A list of all the differentially expressed genes is shown in S1 Dataset. The RNAseq data is available in the Gene Expression Omnibus GSE152727 (https://www.ncbi.nlm.nih.gov/geo/query/acc.cgi?acc=GSE152727).

### Chromatin immunoprecipitation-sequencing (Chip-seq)

Chromatin isolation, chromatin shearing, ChIP, library preparation and Bioanalyzer QC was performed by EpiGentek, details of which are provided in the supplemental material. The ChIPseq data is available in the Gene Expression Omnibus GSE152791 (https://www.ncbi.nlm.nih.gov/geo/query/acc.cgi?acc=GSE152791).

## Supporting information

S1 Fig Sample similarity assessment: The overall similarity among samples were assessed by the euclidean distance between samples. The shorter the distance, the more closely related are the samples.
(TIF)

S2 Fig Different genomic annotations associated with genome wide H3K27me3 peaks in granulosa cells treated with (treatment group) or without (control group) DHT (25nM for 24h). (TIF)

S3 Fig A: HIF1α response elements (HRE) in *Jmjd*3 promoter region. B: Relative expression of *Hif1α* mRNA levels by quantitative PCR in mouse primary GC cultures treated with media (control) or DHT (25nM for 24h). C: Androgens do not suppress HIF1α protein degradation. Time course of HIF1α protein degradation in presence of the translational inhibitor cycloheximide (CHX, 1μM) in mouse primary GC cultures treated with media (control) or DHT (25nM for 24h). D: Jmjd3 enzymatic activity in mouse primary GC cultures treated with media (control) or DHT (25nM for 24h) in presence of flutamide (AR inhibitor, 100nM) or LY294002 (PI3K inhibitor, 10μM). Data are displayed as means ± SEM (n = 3 experiments, for each experiment GCs isolated from 5 mice were pooled together) and normalized to total protein (P ≤ 0.05, * *vs* media and ** vs DHT).
(TIF)

S4 Fig A: Anti-H3K27me3 ChIP-assay in primary mouse GC cultures treated with Jmjd3 siRNA, Hif1α siRNA or non-specific (Nsp) siRNA and stimulated with/without DHT (25nM for 24h) showing H3K37me3 levels within the gene body of *Lhcgr* (+641/+740 bp from TSS), *Fshr* (+379/+488 bp from TSS), *Cyp19a1* (+553/+653 bp from TSS) and *Runx1* (+579/+693 bp from TSS). IgG represents non-specific antibody. Values represent percentage input. Data are displayed as mean ± SEM (n = 3 experiments, for each experiment GCs isolated from 10 mice were pooled together) and * P ≤ 0.05, *vs* Non-specific siRNA. B: H3K27ac levels in primary mouse GCs treated with DHT (25nM for 24h).
(TIF)

S5 Fig Effect of Clioquinol, a HIF1α activator in presence/absence of AR on JMJD3 and H3K27me3 levels. Primary mouse GC cultures were treated with AR specific LNA-siRNA or nonspecific siRNA control, and stimulated with/without DHT (25nM for 24h) in presence or absence of Clioquinol (50**μ**M).
(TIF)

S6 Fig A: Effect of different concentrations of DHT on HIF1α, JMJD3 and H3K27me3 protein levels in primary mouse GC cultures. B: Expression of *Bmp4*, *Angpt1*, *Lhcgr and Mmp2* with respect to different concentrations of DHT in primary mouse GC cultures. Data are displayed as mean ± SEM (n = 3 experiments, for each experiment GCs isolated from 5 mice were pooled together) and normalized to *Rpl19* (P ≤ 0.05, * *vs* media)
(TIF)

S7 Fig A: Antibody List. B: Taqman Gene expression assay Primers. C: Inhibitor List.
(TIF)

S1 Text Materials and Methods. (DOCX)

S1 Dataset List of differentially expressed genes (control vs DHT). (XLSX)

S2 Dataset Genes with H3K27me3 peaks in the gene body of control and DHT-treated samples. (XLSX)

S3 Dataset Genes with promoters overlapping with H3K27me3 peaks in control and DHT-treated samples. (XLSX)

S4 Dataset ChIP primer list. (XLSX)

S5 Dataset Genes with enhancers where H3K27me3 signals are lowered with DHT treatment. (XLSX)

S6 Dataset Androgen-induced DEGS whose enhancers have significantly negative correlated H3K27me3 levels. (XLSX)
