# Supplementary figures and images for: Androgens regulate ovarian gene expression by balancing Ezh2-Jmjd3 mediated H3K27me3 dynamics
Source: PLoS Genet. 2021 Mar 30;17(3):e1009483. doi: 10.1371/journal.pgen.1009483 (PMC8034747; doi:10.1371/journal.pgen.1009483)

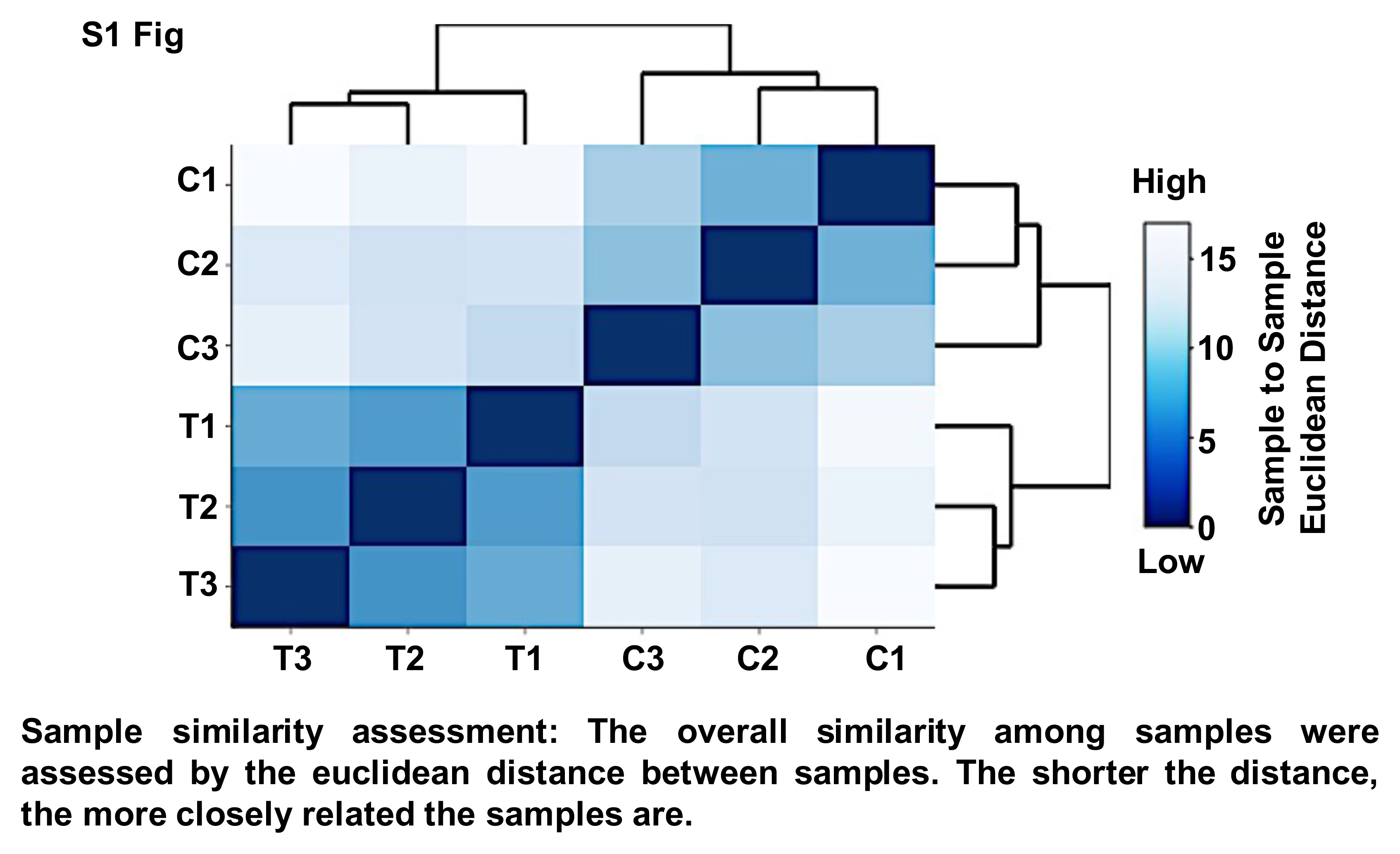

Supplement: S1 Fig — The shorter the distance, the more closely related are the samples. (TIF) [file pgen.1009483.s001.tif]

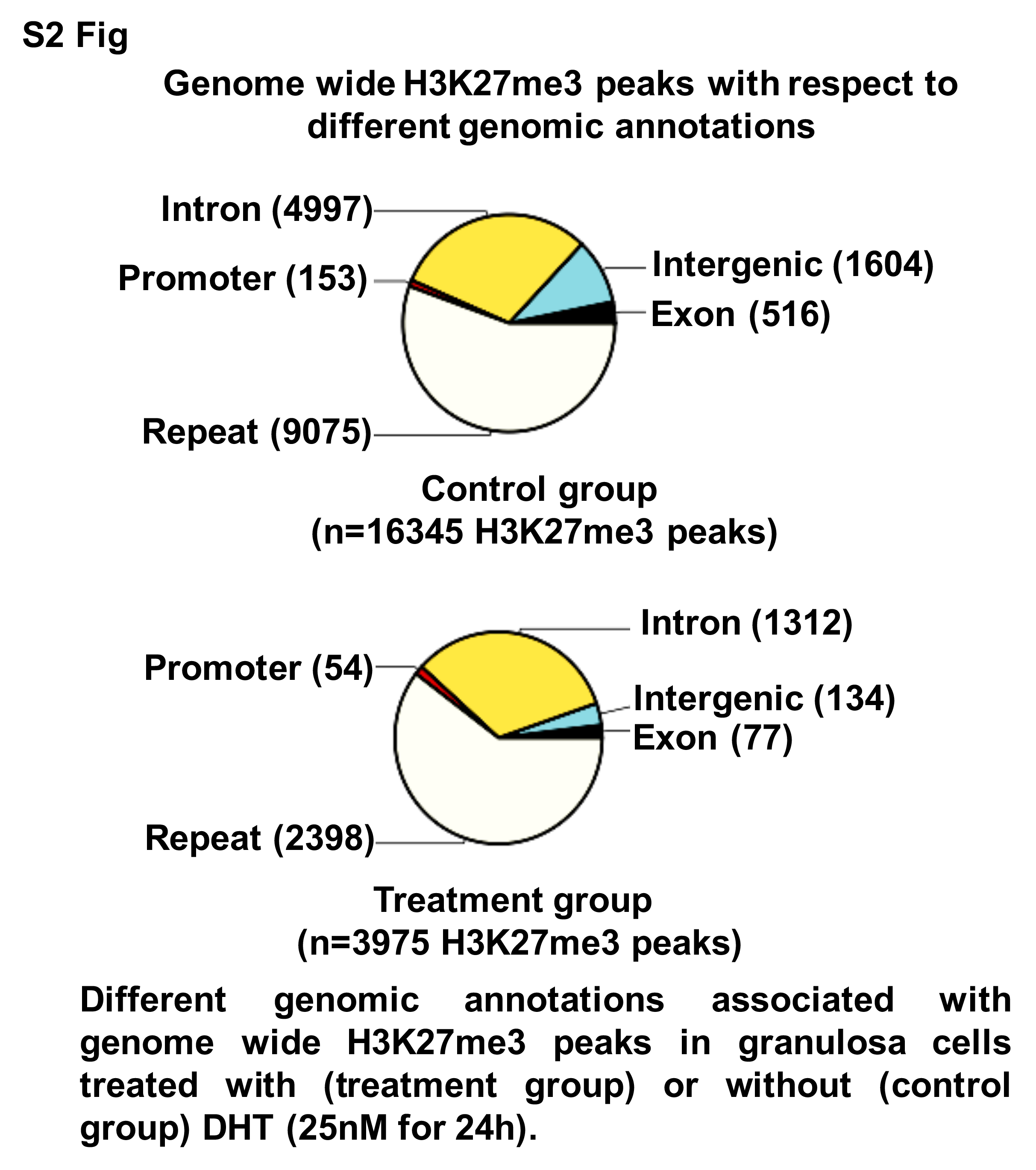

Supplement: S2 Fig — (TIF) [file pgen.1009483.s002.tif]

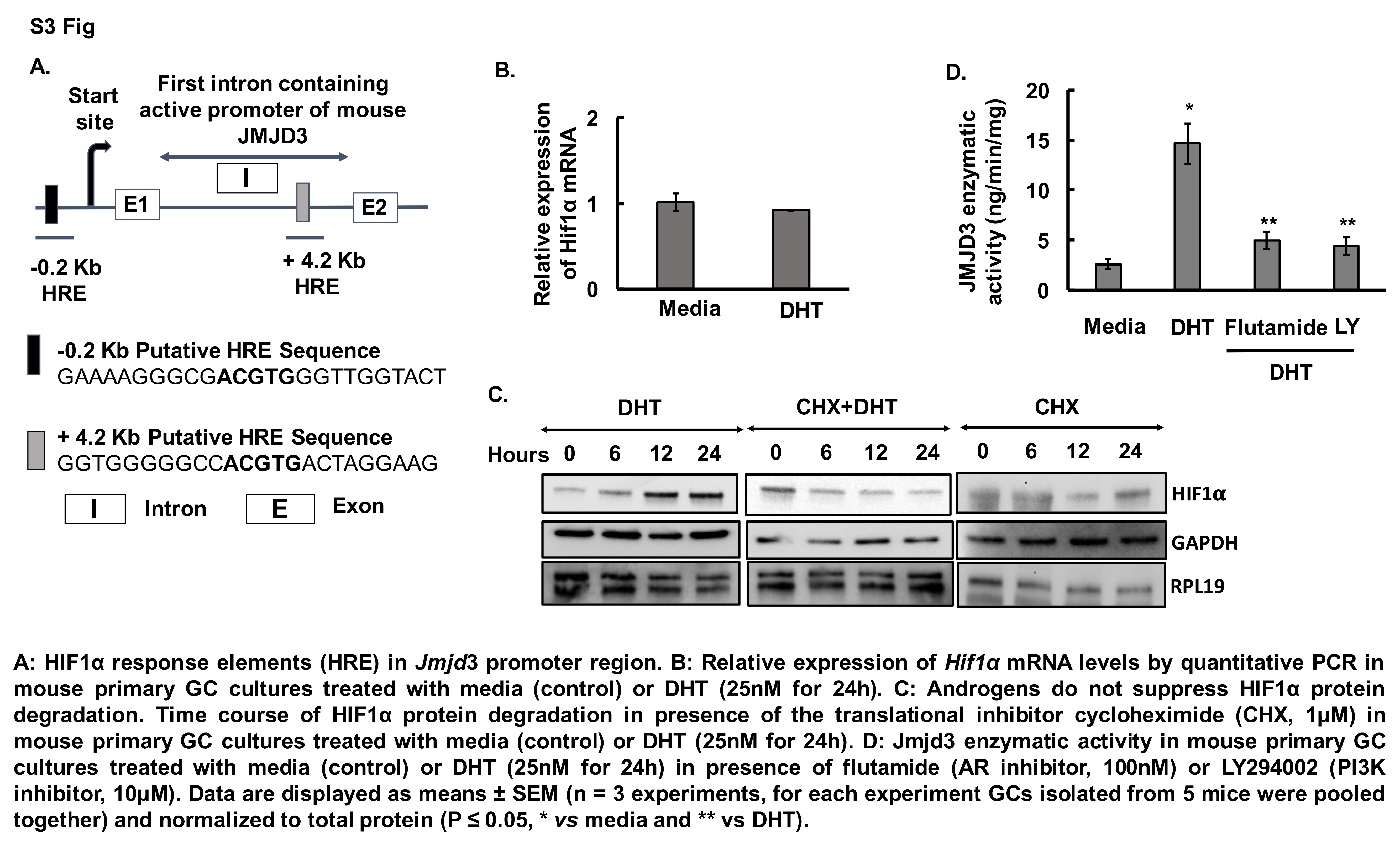

Supplement: S3 Fig — A: HIF1α response elements (HRE) in Jmjd3 promoter region. B: Relative expression of Hif1α mRNA levels by quantitative PCR in mouse primary GC cultures treated with media (control) or DHT (25nM for 24h). C: Androgens do not suppress HIF1α protein degradation. Time course of HIF1α protein degradation in presence of the translational inhibitor cycloheximide (CHX, 1μM) in mouse primary GC cultures treated with media (control) or DHT (25nM for 24h). D: Jmjd3 enzymatic activity in mouse primary GC cultures treated with media (control) or DHT (25nM for 24h) in presence of flutamide (AR inhibitor, 100nM) or LY294002 (PI3K inhibitor, 10μM). Data are displayed as means ± SEM (n = 3 experiments, for each experiment GCs isolated from 5 mice were pooled together) and normalized to total protein (P ≤ 0.05, * vs media and ** vs DHT). (TIF) [file pgen.1009483.s003.tif]

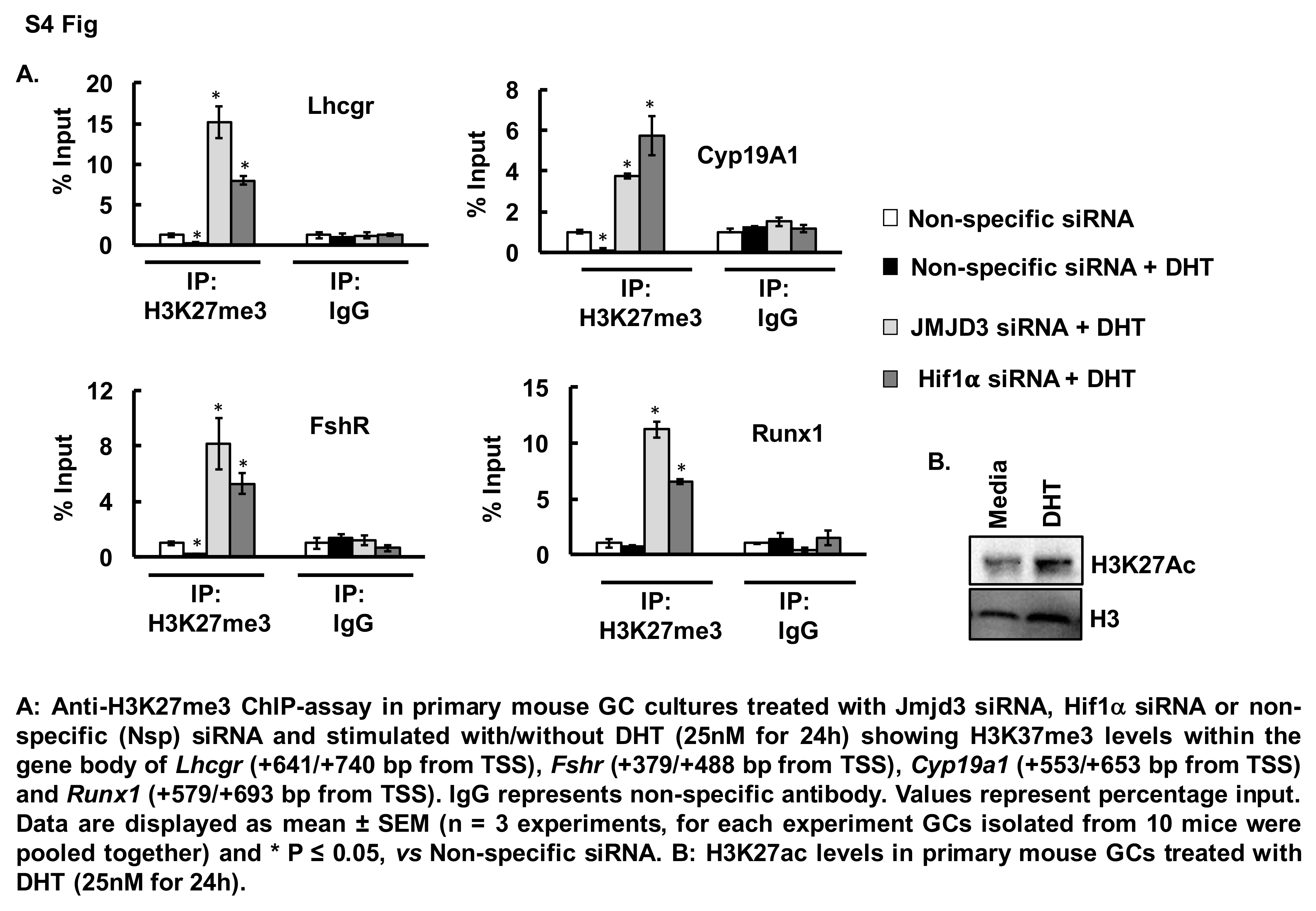

Supplement: S4 Fig — A: Anti-H3K27me3 ChIP-assay in primary mouse GC cultures treated with Jmjd3 siRNA, Hif1α siRNA or non-specific (Nsp) siRNA and stimulated with/without DHT (25nM for 24h) showing H3K37me3 levels within the gene body of Lhcgr (+641/+740 bp from TSS), Fshr (+379/+488 bp from TSS), Cyp19a1 (+553/+653 bp from TSS) and Runx1 (+579/+693 bp from TSS). IgG represents non-specific antibody. Values represent percentage input. Data are displayed as mean ± SEM (n = 3 experiments, for each experiment GCs isolated from 10 mice were pooled together) and * P ≤ 0.05, vs Non-specific siRNA. B: H3K27ac levels in primary mouse GCs treated with DHT (25nM for 24h). (TIF) [file pgen.1009483.s004.tif]

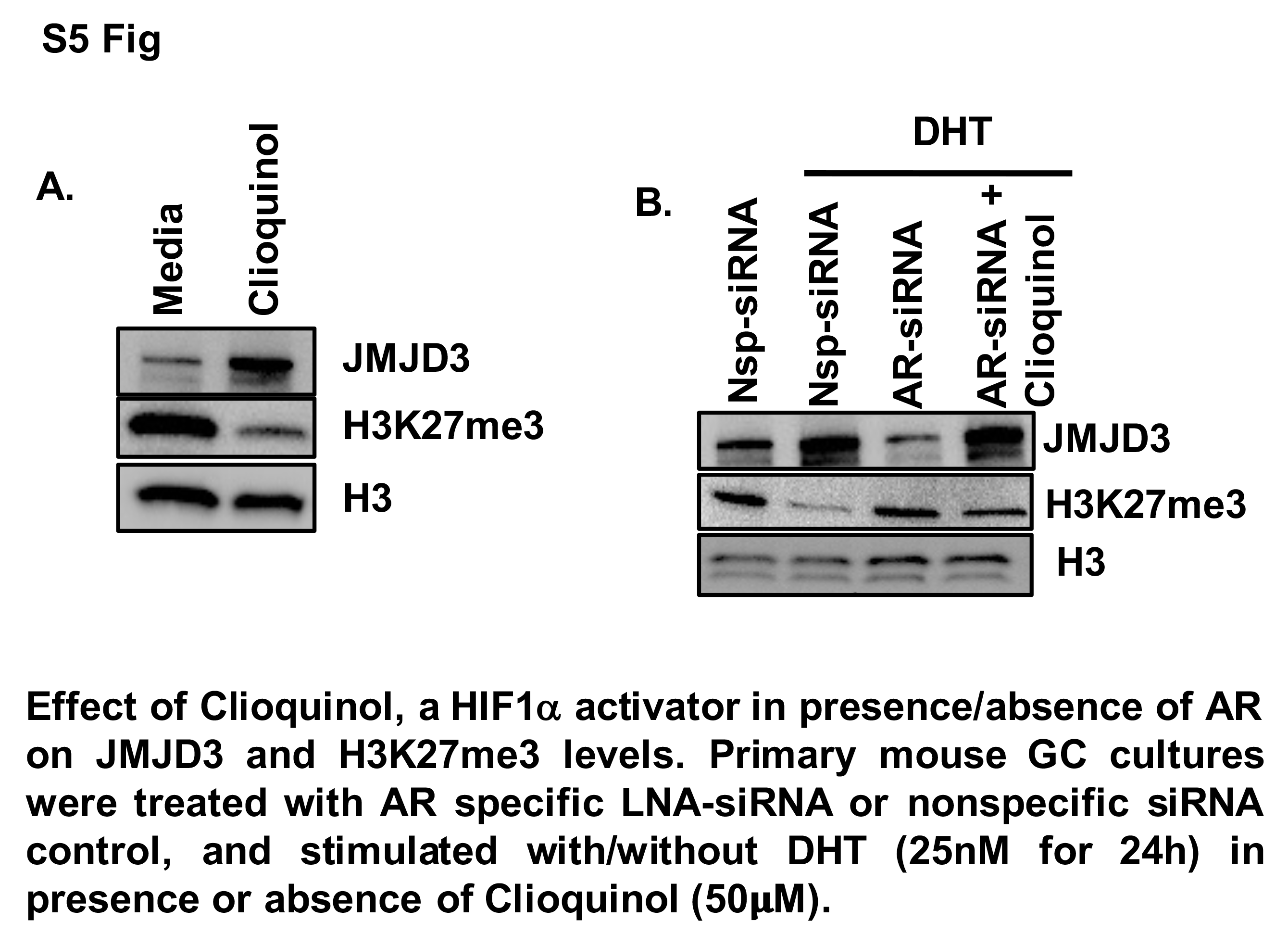

Supplement: S5 Fig — Primary mouse GC cultures were treated with AR specific LNA-siRNA or nonspecific siRNA control, and stimulated with/without DHT (25nM for 24h) in presence or absence of Clioquinol (50μM). (TIF) [file pgen.1009483.s005.tif]

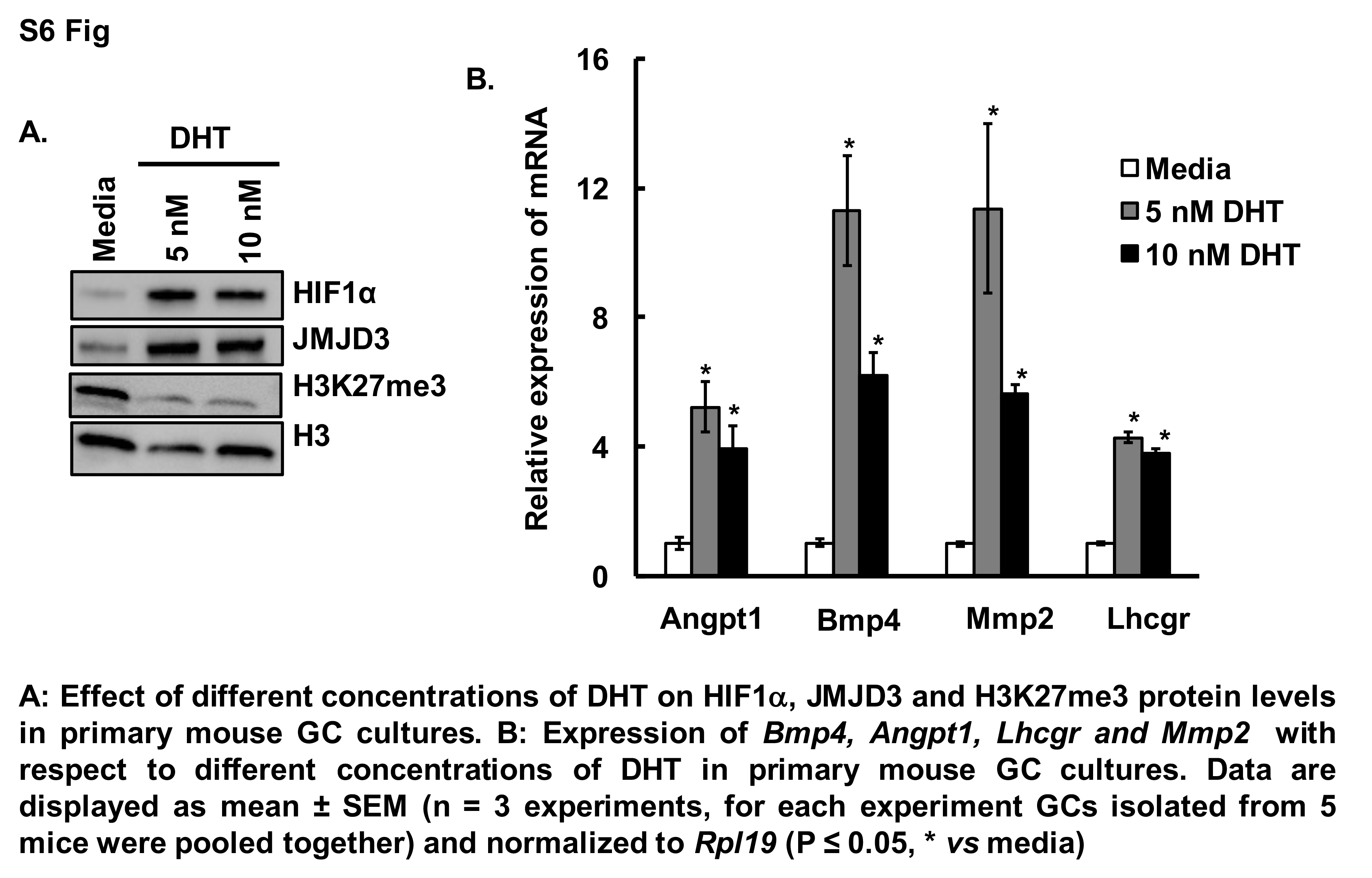

Supplement: S6 Fig — A: Effect of different concentrations of DHT on HIF1α, JMJD3 and H3K27me3 protein levels in primary mouse GC cultures. B: Expression of Bmp4, Angpt1, Lhcgr and Mmp2 with respect to different concentrations of DHT in primary mouse GC cultures. Data are displayed as mean ± SEM (n = 3 experiments, for each experiment GCs isolated from 5 mice were pooled together) and normalized to Rpl19 (P ≤ 0.05, * vs media) (TIF) [file pgen.1009483.s006.tif]

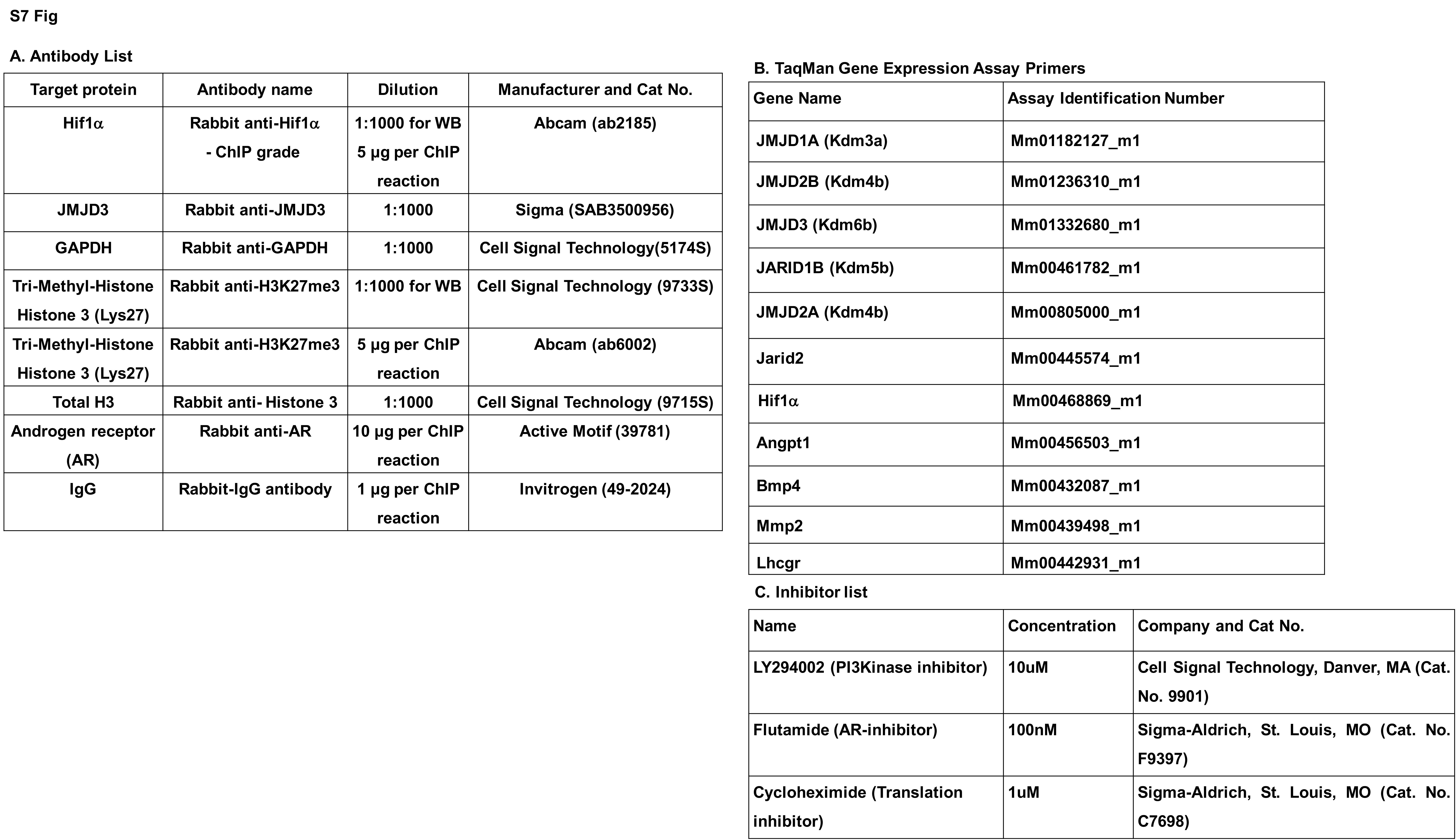

Supplement: S7 Fig — A: Antibody List. B: Taqman Gene expression assay Primers. C: Inhibitor List. (TIF) [file pgen.1009483.s007.tif]
