# Supplementary material for: Androgens regulate ovarian gene expression by balancing Ezh2-Jmjd3 mediated H3K27me3 dynamics
Source: PLoS Genet. 2021 Mar 30;17(3):e1009483. doi: 10.1371/journal.pgen.1009483 (PMC8034747; doi:10.1371/journal.pgen.1009483)
Supplement: S1 Text — (DOCX) [file pgen.1009483.s008.docx]

**S1 Text**

**Materials and Methods**

***Mouse Primary GC culture -*** Estrous cycle was determined by daily vaginal smears as described previously [1-7] and on the day of estrus, granulosa cells (GCs) were isolated by needle puncture under the microscope. Isolated GCs were either snap frozen in liquid nitrogen immediately after isolation or cultured for 48h in DMEM/F-12 medium containing 10% FBS and 1% penicillin and streptomycin. Thereafter, GCs were serum starved for 4h followed by various treatments (as shown in the figures) in serum free media. For each experiment, GCs isolated from 5 animals were pooled together and each experiment was repeated three times. For experiments involving ChIP assay, GCs isolated from 10 animals were pooled together for each experiment. For all experiments with primary mouse GC cultures, cell viability was determined by trypan blue at the time of harvesting. For experiments using inhibitors, cells were pre-treated with the inhibitors for 30 min prior to DHT (dihydrotestosterone, 25nM) stimulation (24h). The concentration of DHT was based on previous studies [1,8].

***Western blot analysis:*** Western blots were performed as described previously [1-7]. A list of antibodies and dilution is provided in the supplemental material (S5A Fig).

***siRNA knockdown experiments****: In vitro:* siRNA-mediated knockdown experiments were performed as described previously [4,6,7] using non-targeting siRNA (Nsp) pool or mouse *Hif1α* / *Jmjd3* siRNA ON-TARGET Plus SMARTpool siRNAs (Dharmacon) according to manufacturer’s instructions. *In vivo*: Ovarian bursal injections were performed as described previously [1,6]. Locked nucleic acid (LNA) modified siRNA targeting *Ar* (ThermoFischer) or non-specific control were mixed with DharmaFECT 1 transfection reagent (Dharmacon) before bursal injection. Each animal (n=15) served as its own control, with one ovary receiving the blocking oligonucleotide and the other the non-specific control. Animals were euthanized 72 hours after injection and GCs isolated from ovaries were processed for ChIP-PCR studies and western blot analysis to detect AR and H3K27me3 levels.

***Chromatin immunoprecipitation* (*ChIP) assay:*** GCs were stimulated with DHT for 24h, and ChIP was performed as described previously [1,4,6,7,9] with rabbit polyclonal anti- HIF1α ChIP grade antibody (Abcam), anti-AR ChIP grade antibody (Active Motif), anti-H3K27me3 ChIP grade antibody (Abcam) or IgG as non-specific control. Quantitative PCR was performed using PerfeCTa SYBR Green Supermix (Quantabio, VWR) with primers designed for (1) two HRE (*Hif1α* receptor binding element) regions on the *Jmjd3* promoter, (2) ARE (androgen receptor binding element) regions on *Bmp4*, *Angpt1*, *Mmp2* and *Lhcgr* and control regions and (3) H3K27me3 sites within the gene body (based on Chip-seq data) of *Lhcgr, Fshr, Cyp19a1* and *Runx1* genes. List of all ChIP primers (S4 Dataset) and antibodies (S7A Fig) are provided in the supplemental data.

***RNA isolation, Quantitative Real Time PCR* (qRT-PCR) *and RNA-seq:*** Total RNA was isolated by using E.Z.N.A Total RNA kit (Omega) according to the manufacturer's instructions. 1μg of RNA was used for all the qRT-PCR reactions. The expression assay primers are shown in S7B Fig. Each target gene was normalized to *Rpl19*. The mRNA expression was analyzed by ∆/∆ Ct method.

For RNA-seq, total RNA was extracted from GCs (GCs from 5 animals were pooled together to make n=1 and three such pooled samples/treatment were used for the RNA-seq studies) using Qiagen RNeasy Plus Universal mini kit followed by Manufacturer’s instructions (Qiagen, Hilden, Germany). RNA samples were quantified using Qubit 2.0 Fluorometer (Life Technologies, Carlsbad, CA, USA) and RNA integrity was checked using Agilent TapeStation 4200 (Agilent Technologies, Palo Alto, CA, USA). RNA integrity number (RIN) for all the samples was between 9.7 to 10. RNA sequencing libraries were prepared using 500ng RNA, and the NEBNext Ultra RNA Library Prep Kit for Illumina using manufacturer’s instructions (NEB, Ipswich, MA, USA). The sequencing library was validated on the Agilent TapeStation (Agilent Technologies, Palo Alto, CA, USA), and quantified by using Qubit 2.0 Fluorometer (Invitrogen, Carlsbad, CA) as well as by quantitative PCR (KAPA Biosystems, Wilmington, MA, USA). The sequencing libraries were clustered on a single lane of a flowcell. After clustering, the flowcell was loaded on the Illumina HiSeq instrument (4000 or equivalent) according to manufacturer’s instructions. The samples were sequenced using a 2x150bp Paired End (PE) configuration. The total number of reads/sample were 45-50 million. Image analysis and base calling were conducted by the HiSeq Control Software (HCS). Raw sequence data (.bcl files) generated from Illumina HiSeq was converted into fastq files and de-multiplexed using Illumina's bcl2fastq 2.17 software. One mismatch was allowed for index sequence identification.

***Bioinformatics analysis for RNA-seq:*** Raw data quality was judged based on Illumina’s Q score, which represents the error rate at each base, built on a log10 score. Thereafter, sequence reads were trimmed to remove possible adapter sequences and nucleotides with poor quality using Trimmomatic v.0.36. The trimmed reads were mapped to the reference *Mus musculus* GRCm38 genome available on ENSEMBL using the STAR aligner v.2.5.2b. The STAR aligner uses a splice aligner that detects splice junctions and incorporates them to help align the entire read sequences. BAM files were generated as a result of this step. Unique gene hit counts were calculated by using feature Counts from the Subread package v.1.5.2. Only unique reads that fell within exon regions were counted. After extraction of gene hit counts, the gene hit counts table was used for downstream differential expression analysis. Using DESeq2 R package, differentially expressed genes (DEGs) were identified between control (n = 3) and DHT-treatment (n = 3). The heat maps were constructed using rlog transformed values obtained from RNA-seq data followed by z-normalization. The Wald test was used to generate p-values and Benjamini-Hochberg test for adjusted p-value. Genes with adjusted p-values ≤ 0.05 and absolute log2 fold changes > 1 were called as differentially expressed genes for each comparison***.*** For Gene ontology analysis, significantly differentially expressed genes were clustered by their gene ontology and the enrichment of gene ontology terms were tested using Fisher exact test (GeneSCF v1.1-p2). Pathways with p-values ≤ 0.05 were considered significant.

***Chromatin immunoprecipitation-sequencing (Chip-seq):*** Granulosa cells were collected from 72 mice and plated in six petri dishes (each with at least 1×10^7^ cells). Three plates were used as control while the other three were DHT treated. Chromatin isolation, chromatin shearing, ChIP, library preparation and Bioanalyzer QC was performed by EpiGentek, NY. Chromatin was isolated using the ChromaFlash Chromatin Extraction Kit (EpiGentek, Cat. #P-2001) and sheared using the EpiSonic 2000 Sonication System (EpiGentek, Cat. #EQC-2000). DNA fragment size distribution was determined to be 100 to 300 bp by Agilent Bioanalyzer analysis. The concentration of DNA following ChIP was between 3.5-16.3 ng. Library Preparation was done by DNA end polishing and adaptor ligation, library amplification using indexed primers and library purification. Library was verified on a Bioanalyzer and KAPA qPCR Library Quantification.

***Bioinformatics analysis for ChIP-seq:*** FastQC (version 0.11.7) was used for quality controls of ChIP-seq datasets, including 3 replicates for control and 3 replicates for DHT treated GCs. The ChIP-seq reads were mapped to the reference mouse genome (mm9) using Bowtie2 (version 2.3.4), and only uniquely mapped reads were used for subsequent analyses. MACS2 (version 2.1.1) was then applied to identify signal peaks of H3K27me3 signals, in broad peak calling mode since H3K27me3 distribution along the genome is more diffusive and demonstrate broader peaks. PCR duplicates were removed in peak calling. Peaks with q-value ≤ 0.05 were used as the significant signal peaks. Peaks in control samples and treatment samples are center-aligned separately with +/-10kb flanking regions. The flanking regions of each peak were further divided into 100bp bins and the normalized H3K27me3 ChIP-seq read densities for each bin were plotted. The genomic locations of significant H3K27me3 peaks in controls and DHT treated samples were then compared to gene annotations to identify genes overlapping with H3K27me3 peaks in gene bodies. Promoters (+/- 1kb from transcription start sites) were also compared with H3K27me3 peaks. Gene promoters overlapping with control-specific H3K27me3 peaks were center-aligned with +/- 1kb flanking regions, and the average peak densities per 50bp bins were plotted. Similar peak densities were plotted for gene promoters overlapping with treatment-specific H3K27me3 peaks. For differentially expressed genes that also contain H3K27me3 peaks in gene bodies, their gene bodies were divided into 10 bins, starting from transcription start sites (TSS) to transcription end sites (TES), where every bin represented 10% of the specific gene’s body. The number of H3K27me3 peaks in each bin were then calculated for every gene, based on H3K27me3 signals from control samples and treatment samples.

A combined chromatin interaction dataset, including Hi-C and Capture-C, were used to identify candidate distal enhancers that can interact with promoters of differentially expressed genes. Hi-C datasets include: GSE81503, GSE82144, GSE119171, GSE121753, and GSE63525. Capture-C dataset includes GSE81503. For the long-range chromatin interactions profiled in these dataset, they were first compared with promoters of differentially expressed genes and a subset of chromatin interactions was then identified, if one of the interacting anchors overlapped with promoters. For each interaction in this subset, the other interacting anchor of the interaction that did not overlap with promoters were identified as candidate distal enhancers that may regulate the gene. The identified enhancer regions from different chromatin interaction datasets were then combined together. To purify false positives of enhancers, we calculated the Pearson correlations between the gene’s expression levels and the enhancer’s H3K27me3 signal levels across the 6 samples. The lengths of enhancers and the library sizes of different samples were normalized for H3K27me3 signals. Therefore, for each enhancer-gene pair, an activity correlation was calculated. Only enhancer-gene pairs with correlations < -/+0.4 were considered as true regulatory pairs and the corresponding enhancers were used for subsequent analysis. For each differentially expressed gene with distal interacting enhancers, we generated the distribution of the numbers of enhancers co-regulating the same genes. We also calculated the distribution of the distances between gene promoters and distal interacting enhancers.

Motif enrichment analysis was applied on the identified enhancers using MEME (version 5.0.4). Enhancers were classified into two groups: the first group of enhancers interact with promoters of up-regulated genes and the second group of enhancers interact with promoters of down-regulated genes. For each group of enhancers, the top five enriched sequence motifs were identified using MEME. TomTom (version 5.0.4) was then applied on the top-enriched motifs to identify the corresponding transcription factors, based on transcription factor motif annotations in “HOCOMOCOv11_full_MOUSE_mono_meme_format.meme” from the MEME suite. The matched transcription factors with E-value < 0.1 were then identified as the candidate factors associated with epigenetic changes in distal enhancers.

***JMJD3 enzymatic assay:*** JMJD3 enzymatic activity was measured using JMJD3/UTX Demethylase Activity/Inhibition assay kit (colorimetric) from Epigentek as per the manufacturer’s instruction. Data was normalized to total protein.

***Statistical analysis:*** Statistical analysis was performed using GraphPad Prism version 8.0.2 (GraphPad Software). Statistical comparisons were made by paired t test (for comparing two groups) or a one-way ANOVA followed by multiple comparison test and results with P ≤ 0.05 were considered significant.

**Supplemental References**

1. Ma, X., Hayes, E., Biswas, A., Seger, C., Prizant, H., Hammes, S. R., and Sen, A. (2017) Androgens Regulate Ovarian Gene Expression Through Modulation of Ezh2 Expression and Activity. *Endocrinology* **158**, 2944-2954

2. Ma, X., Hayes, E., Prizant, H., Srivastava, R. K., Hammes, S. R., and Sen, A. (2016) Leptin-induced CART (Cocaine-and Amphetamine-Regulated Transcript) is a novel intra-ovarian mediator of obesity-related infertility in females. *Endocrinology*, en20151750

3. Hayes, E., Kushnir, V., Ma, X., Biswas, A., Prizant, H., Gleicher, N., et al. (2016) Intra-cellular mechanism of Anti-Mullerian hormone (AMH) in regulation of follicular development. *Mol Cell Endocrinol* **433**, 56-65

4. Roy, S., Gandra, D., Seger, C., Biswas, A., Kushnir, V. A., Gleicher, N., et al. (2018) Oocyte-Derived Factors (GDF9 and BMP15) and FSH Regulate AMH Expression Via Modulation of H3K27AC in Granulosa Cells. *Endocrinology* **159**, 3433-3445

5. Sen, A., O'Malley, K., Wang, Z., Raj, G. V., Defranco, D. B., and Hammes, S. R. (2010) Paxillin regulates androgen- and epidermal growth factor-induced MAPK signaling and cell proliferation in prostate cancer cells. *J Biol Chem* **285**, 28787-28795

6. Sen, A., Prizant, H., Light, A., Biswas, A., Hayes, E., Lee, H. J., et al. (2014) Androgens regulate ovarian follicular development by increasing follicle stimulating hormone receptor and microRNA-125b expression. *Proc Natl Acad Sci U S A* **111**, 3008-3013

7. Sinha, N., Biswas, A., Nave, O., Seger, C., and Sen, A. (2019) Gestational Diabetes Epigenetically Reprograms the Cart Promoter in Fetal Ovary, Causing Subfertility in Adult Life. *Endocrinology* **160**, 1684-1700

8. Sen, A., Prizant, H., and Hammes, S. R. (2011) Understanding extranuclear (nongenomic) androgen signaling: what a frog oocyte can tell us about human biology. *Steroids* **76**, 822-828

9. Sinha, N., Roy, S., Huang, B., Wang, J., Padmanabhan, V., and Sen, A. (2020) Developmental Programming: Prenatal Testosterone-induced Epigenetic Modulation and its Effect on Gene Expression in Sheep Ovary. *Biol Reprod*
